# Inflammatory Drug-Resistant Epilepsy Index (IDREI) as a Molecular Compound Biomarker in Focal Epilepsies

**DOI:** 10.3390/biom15070914

**Published:** 2025-06-22

**Authors:** Maria José Aguilar-Castillo, Guillermo Estivill-Torrús, Guillermina García-Martín, Pablo Cabezudo-García, Yolanda López-Moreno, Jesús Ortega-Pinazo, Teresa Ramírez-García, Nicolas Lundahl Ciano-Petersen, Pedro Jesus Serrano-Castro

**Affiliations:** 1 Servicio de Análisis Clínicos, Hospital Regional Universitario de Málaga, 29010 Málaga, Spain; maijo.aguilarcastillo@gmail.com; 2 Instituto de Investigación Biomédica de Málaga y Plataforma de Nanomedicina-IBIMA Plataforma BIONAND, 29590 Málaga, Spain; guillerminagmartin@gmail.com (G.G.-M.); pablocabezudo@gmail.com (P.C.-G.); yolandalopm@gmail.com (Y.L.-M.); jesus.ortega@ibima.eu (J.O.-P.); terramgar@gmail.com (T.R.-G.);; 3 Servicio de Neurología, Unidad de Neurociencias, Hospital Regional Universitario de Málaga, 29010 Málaga, Spain; 4 Red Andaluza de Investigación Clínica y Traslacional en Neurología (Neuro-RECA), 29010 Málaga, Spain; 5 Departamento de Medicina y Dermatología, Universidad de Málaga, 29010 Málaga, Spain

**Keywords:** epilepsy, neuroinflammation, biomarkers, epileptogenesis, drug-resistant epilepsy

## Abstract

Background: There is growing evidence that neuroinflammation is involved in epileptogenesis. Identifying its biomarkers can be important for distinguishing epilepsy patients from healthy individuals and differentiating well-controlled epilepsy from drug-resistant epilepsy (DRE). Methods: An observational case-control study at Malaga’s Regional University Hospital involved epilepsy patients divided into three groups: healthy controls (HC), seizure-free epilepsy (SFE), and DRE. Demographic and clinical data and plasmatic and/or CSF levels of 24 different inflammation-related molecules were collected for each patient and were analyzed through univariate and multivariate analysis. Results: The study included 68 patients: 38 in the DRE group, 14 in the SFE group, and 16 in the HC group. A new Inflammatory Drug-Resistant Epilepsy Index (IDREI) was created using key variables with significant or trending significance. This index combined pro-inflammatory mediators (ICAM-1 and NfL) and anti-inflammatory factors (IL-10 and IL-4), showing statistical significance (*p* = 0.002). ROC curve analysis for the IDREI gave an AUC of 0.731 (95% CI: 0.608–0.854). A multivariate logistic regression model’s ROC analysis resulted in a higher AUC of 0.891 (95% CI: 0.791–0.991). Conclusions: The IDREI molecular index shows promise in predicting epilepsy and drug-resistant epilepsy (DRE). Additional prospective studies are required to assess its clinical utility.

## 1. Introduction

Epilepsy is the most common chronic neurological disease, affecting over 50 million people worldwide, according to the World Health Organization (WHO). The prevalence in developed countries ranges from 3.2 to 7.8 cases per 1000 inhabitants [1], whereas it is significantly higher in developing nations, largely due to a greater number of epilepsy cases stemming from infectious causes [2,3,4].

Epilepsy exhibits significant etiological heterogeneity. In the latest ILAE classification of epilepsies [5], six different etiological categories were recognized: structural, genetic, infectious, metabolic, immune, or unknown. Among the structural causes, the most frequent corresponds to focal cortical dysplasia, hippocampal sclerosis, slow-growing glial tumors, or vascular malformations. On the other hand, less frequent etiologies, including those of genetic, metabolic, or immunity-related origin, have acquired wide recognition in recent times [6,7,8].

Over the past two decades, there has been growing evidence of both clinical and basic studies providing strong support for the conclusion that neuroinflammation is involved in epileptogenesis and that it does so in a transversal way, regardless of the underlying etiology [9,10,11]. Chronic inflammation in the brain can disrupt neuronal excitability, contribute to neuronal damage, and potentially alter drug transport and efficacy [12].

This phenomenon is not exclusive to epilepsy. Multiple neurological diseases show a pathogenic basis strongly influenced by neuroinflammation, where it is, therefore, possible to define certain pro- or anti-inflammatory biomarkers that may relate to early diagnosis, prognosis, or response to treatments. Perhaps the most paradigmatic example is multiple sclerosis [13,14], a condition in which various neuroinflammation and/or neurodegeneration biomarkers have been developed in both plasma and cerebrospinal fluid (CSF). However, it is not the only example, as other conditions, such as autoimmune encephalitis, neuromyelitis optica, opsoclonus-myoclonus syndrome, Rasmussen’s encephalitis, or neuropsychiatric lupus erythematosus, also have studies addressing the role of molecular biomarkers related to neuroinflammation in both plasma and CSF [15].

With the development of personalized and precision neurology, it is essential to expand our understanding of the basic mechanisms of epileptogenesis, with special interest in those related to neuroinflammatory mechanisms. This knowledge can lead to the identification of diagnostic or prognostic neuroinflammatory biomarkers and potentially provide therapeutic targets [6].

The identification of reliable neuroinflammatory biomarkers capable of distinguishing individuals with epilepsy from healthy controls and, more crucially, differentiating between patients with well-controlled epilepsy and those with drug-resistant epilepsy (DRE) presents substantial clinical potential. Since early detection of DRE allows for prompt consideration of non-pharmacological treatments like neuromodulation or epilepsy surgery, identifying inflammatory biomarkers that indicate refractoriness is essential for the proper management of individuals with this condition [16,17].

Recent studies have aimed to identify these biomarkers, but the findings remain inconclusive. Our recent systematic review identified several molecules as potential biomarkers for clinical use; however, none have achieved a high level of scientific evidence [18]. Therefore, the role of these biomarkers in assessing the degree of refractoriness or diagnosing epilepsy remains unknown.

This case-control study aims to investigate and compare the levels of 24 selected molecules implicated in neuroinflammation across three distinct groups: healthy controls (HC), patients with seizure-free epilepsy (SFE), and patients with DRE. By employing univariate and multivariate statistical analyses, as well as receiver operating characteristic (ROC) curve analysis, we seek to define a potential biomarker profile capable of accurately identifying patients with epilepsy and, crucially, distinguishing those with DRE. The findings of this study could contribute to a better understanding of the role of neuroinflammation in epilepsy progression and provide valuable insights for the development of diagnostic and prognostic tools in this challenging condition.

## 2. Materials and Methods

### 2.1. Study Design

An analytical observational case-control study was conducted involving patients with epilepsy from the Epilepsy Unit of the Regional University Hospital of Malaga.

### 2.2. Patients

Patients were recruited consecutively and were divided into three groups.

- **HC group**: Individuals with no history of epilepsy or other known chronic neurological or inflammatory diseases.
- **SFE group:** Patients with a confirmed diagnosis of epilepsy who exhibited adequate seizure control (absence of seizures in the past 12 months) under pharmacological treatment.
- **DRE group:** Patients with a confirmed diagnosis of DRE, defined as those who failed to achieve adequate seizure control despite treatment with at least two appropriate anti-seizure medications (ASM) used at tolerated doses for an adequate period (according to the International League Against Epilepsy—ILAE guidelines) [19].

### 2.3. Patients and Sampling

The inclusion and exclusion criteria for each group were as follows:

- **HC group:**
  ▪ **Inclusion:** Absence of a diagnosis of epilepsy, neurological diseases, chronic inflammatory, autoimmune, or active infectious diseases.
  ▪ **Exclusion:** First-degree family history of epilepsy, presence of any significant medical condition that could affect neuroinflammation biomarkers, chronic use of immunomodulatory or anti-inflammatory drugs.
- **SFE group:**
  ▪ **Inclusion:** Confirmed diagnosis of epilepsy according to ILAE criteria, absence of epileptic seizures in the past 12 months.
  ▪ **Exclusion:** Presence of progressive epileptic syndromes, evidence of underlying active encephalopathy, significant neurological or inflammatory comorbidities unrelated to epilepsy, and recent changes in antiepileptic medication.
- **DRE group:**
  ▪ **Inclusion:** Confirmed diagnosis of DRE according to the ILAE definition, current treatment with at least two antiepileptic drugs.
  ▪ **Exclusion:** Presence of progressive epileptic syndromes, evidence of underlying active encephalopathy, significant neurological or inflammatory comorbidities unrelated to epilepsy.

All participants or their legal representatives provided written informed consent before their inclusion in the study, in accordance with the ethical guidelines of the Declaration of Helsinki and approved by the Research Ethics Committee of Málaga (Protocol code 1228-N-23, date of approval: 25 July of 2023).

### 2.4. Sample Collection and Biomarker Determination

Plasma and cerebrospinal fluid (CSF) samples were collected from each participant under standardized conditions. Blood samples were collected by standard venipuncture in BD Vacutainer^®^ Citrate Tubes containing 0.109 M (3.2%) sodium citrate (9:1 blood to anticoagulant ratio) (BD, Franklin Lakes, NJ, USA). Plasma were isolated within a maximum of two hours post-collection by centrifugation at room temperature for 10 min at 2500× *g*. Samples were stored at −80 °C until further analysis. CSF samples were collected via lumbar puncture and transferred into polypropylene tubes. Within a maximum of two hours post-collection, samples were centrifuged at room temperature for 10 min at 900× *g*. The resulting supernatant was then aliquoted and stored at −80 °C prior to comprehensive assessment.

The levels of the following molecules in plasma were determined as follows:

- Pro-inflammatory biomarkers (20): MIP-1α (Macrophage Inflammatory Protein 1-alpha), IL-1β (Interleukin-1 beta), IP-10 (Interferon gamma-induced Protein 10 or CXCL10), IL-8 (Interleukin-8), IL-12 (Interleukin-12), IL-17A (Interleukin-17A), IL-33 (Interleukin-33), IFN-γ (Interferon gamma), GM-CSF (Granulocyte-Macrophage Colony-Stimulating Factor), TNF-α (Tumor Necrosis Factor-alpha), MIP-1β (Macrophage Inflammatory Protein 1-beta), IFN-α (Interferon-alfa), MCP-1 (Monocyte Chemoattractant Protein-1), P-Selectin (CD62P), IL-1α (Interleukin-1 alfa), ICAM-1 (Intercellular Adhesion Molecule-1), E-Selectin (CD62E), sTNF-RII (soluble tumor necrosis factor receptor TNF-RII), TLR4 (Toll-Like Receptor 4), HMGB1 (High Mobility Group Box 1).
- Anti-inflammatory biomarkers (3): IL-4 (Interleukin-4), IL-10 (Interleukin-10), and IL-13 (Interleukin-13).
- Dual function (2): IL-33, sTNF-RII (depending on the context).

Furthermore, to include a biomarker associated with neurodegeneration and indirectly with neuroinflammation, we opted to determine the levels of Neurofilament Light Chain (NfL) in cerebrospinal fluid (CSF).

The selection of biomarkers included was decided based on data from previous literature.

Inflammatory and anti-inflammatory biomarkers in plasma samples were analyzed using the ProcartaPlex™ Human Inflammation Panel, 20 plex kit (catalog number: EPX200-12185-901; Thermo Fisher Scientific Inc., Waltham, MA, USA). Detection was carried out with xMAP^®^ technology on the Luminex MAGPIX^®^ instrument (Diasorin S.p.A., Saluggia, VC, Italy), and data were processed using xPONENT^®^ 4.3 software (Diasorin S.p.A., Saluggia, VC, Italy).

ELISA kits from ElabScience^®^ (Houston, TX, USA) were used to measure HMGB1 and TLR4 (catalog numbers: E-EL-H1554 and E-EL-H1539, respectively). Another ELISA kit, the Human sTNF RII/TNFRSF1B Quantikine^®^ ELISA Kit (catalog number: DRT200; R&D Systems, Inc., Bio-Techne Ltd., Minneapolis, MN, USA), was used to detect sTNFR II. Neurofilament Light Chain (NfL) levels in cerebrospinal fluid were measured using the UmanDiagnostics NF-light™ ELISA RUO kit (catalog number: 10-7002, Quanterix Corp., Billerica, MA, USA).

Samples were diluted according to the requirements of the corresponding kits. Duplications were made for each sample.

Analyses were performed following the manufacturers’ standard protocols, with appropriate quality controls, to ensure the accuracy and reliability of the results.

The comprehensive technical procedure for biomarker determination can be found in the Supplementary Material accompanying this article.

### 2.5. Demographic and Clinical Data

Sex, age, epilepsy onset, duration of epilepsy, type of seizures, magnetic resonance imaging (MRI) findings, EEG findings, etiology, frequency of seizures, number of anti-seizure drugs, number of actual Anti-seizure medication (ASM), and mechanism of action of ASM were retrospectively collected for each of the patients referred to the time of sample extraction.

### 2.6. Statistical Analysis

Statistical analysis was performed using SPSS 25.0 statistical software and included the following:

- **Descriptive Analysis:** Descriptive statistics (mean, standard deviation for continuous variables; frequencies and percentages for categorical variables) were calculated to characterize the three study populations. Normality tests (e.g., Shapiro–Wilk) were used to determine the distribution of continuous variables.
- **Univariate Analysis:** The concentrations of each of the 24 determinations of biomarkers were compared between the three groups (HC, SFE, DRE) using appropriate statistical tests. For continuous variables with normal distribution, analysis of variance (ANOVA) followed by post-hoc tests (Bonferroni) for pairwise comparisons was used. For continuous variables without normal distribution, the Kruskal–Wallis test followed by Dunn’s post-hoc tests with Bonferroni correction was used. For categorical variables, the chi-square test or Fisher’s exact test was used, as appropriate.
- **Index Generation:** Molecules that achieved statistical significance in the multivariate analysis, as well as those with results approaching significance, were selected. The index was designed to encompass both pro-inflammatory and anti-inflammatory factors.
- **ROC Curve Analysis:** To evaluate the diagnostic potential of biomarkers that showed significant differences in the univariate and/or multivariate analysis to discriminate between groups, receiver operating characteristic (ROC) curve analyses were performed. The area under the curve (AUC) with its 95% CI, sensitivity, and specificity for different cut-off points were calculated. The optimal cut-off point was determined using Youden’s index.
- **Multivariate Analysis:** Multinomial logistic regression models were constructed to identify which biomarkers were independently associated with membership in the epilepsy groups (SFE and DRE) compared to the healthy control group, adjusting for potential confounding variables.

A *p*-value of < 0.05 was considered statistically significant for all analyses.

## 3. Results

### 3.1. Descriptive Analysis

A cohort of 68 patients was enrolled and stratified into three distinct groups: the DRE group (*n* = 38), the SFE group (*n* = 14), and the HC group (*n* = 16).

Baseline demographic and clinical parameters, including age, sex, epilepsy duration, epilepsy onset, seizure type, MRI findings, etiology, and the pharmacological mechanisms of action of anti-seizure medications (ASMs), were found to be homogeneous across the groups.

Conversely, statistically significant disparities were identified in interictal electroencephalogram (EEG) findings, seizure frequency, and the number of ASMs (detailed in Table 1).

### 3.2. Univariate Analysis

A univariate analysis was performed to quantify 23 molecules in plasma and 1 molecule in CSF. Descriptive results, expressed as mean (±SD) and median (IQR), along with statistical significance values, are detailed in Table 2. Statistical comparisons were conducted using ANOVA for normally distributed data and the Kruskal–Wallis test for non-normally distributed data.

While most analyses showed no significant differences, a notable exception was plasma ICAM-1, which exhibited a statistically significant difference (*p* = 0.0305) when comparing medians across the three groups.

### 3.3. Index Generation

The IDREI was constructed using a selection of variables from the univariate analysis that demonstrated either statistical significance or a trend toward significance. This approach is intended to reflect the “inflammatory state” in the patient by incorporating both pro-inflammatory factors, specifically ICAM-1 (*p* = 0.0035) and NfL (*p* = 0.145), and anti-inflammatory factors, namely IL-10 (*p* = 0.116) and IL-4 (*p* = 0.169).

The IDREI was formulated as follows:

$$\frac{ICAM1\times NfL}{IL10\times IL4\times 100000}$$

All molecular concentrations were expressed in pg/mL. The newly generated IDREI variable underwent a non-parametric comparison of medians using the Kruskal–Wallis test, which yielded a statistically significant result (*p* = 0.002). Detailed results for the IDREI are presented in Table 3.

To ensure the validity of the constructed index, we assessed multicollinearity among its constituent variables. This was achieved by calculating the Variance Inflation Factor (VIF). An index is generally considered valid if the VIF is less than 5, a condition that was met in our analysis (Table 4).

### 3.4. ROC Curve Analysis

**Figure 1:**
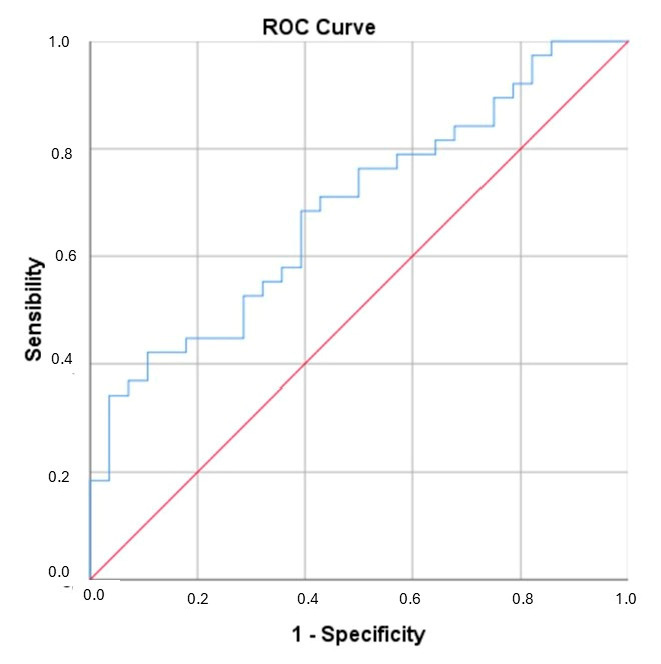
ROC for ICAM-1. AUC: 0.706 (CI: 0.581–0.830).

**Figure 2:**
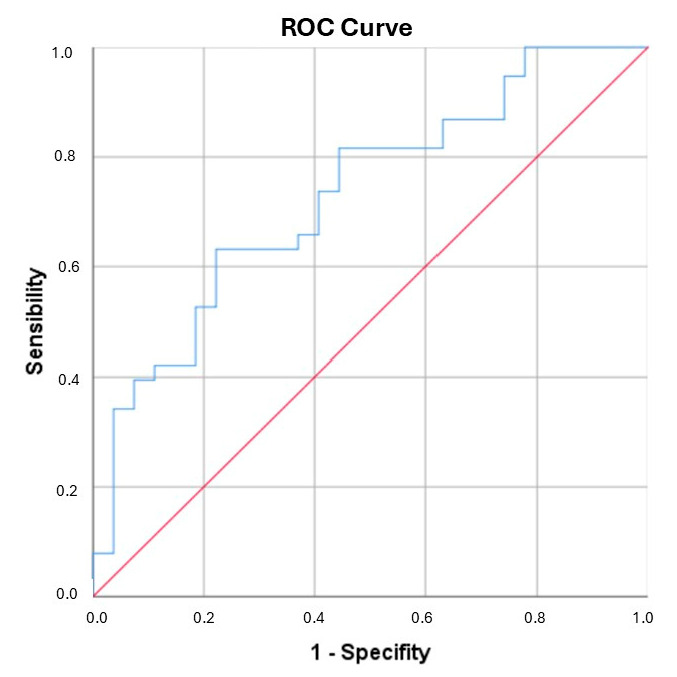
ROC for IDREI. AUC: 0.731 (CI: 0.608–0.854). The different colors reflex the nature of the curve. Red reflect the expected curve if there is no diagnostic capacity. Blue color reflects our results.

**Figure 3:**
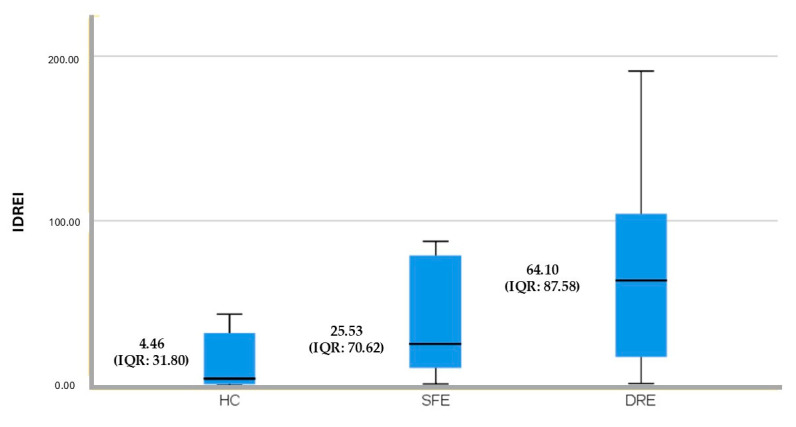
Box plot illustrating the distribution of IDREI values across the different study groups.

A ROC curve analysis was performed for ICAM-1 and the newly generated variable (IDREI) using the DRE group as a reference category (Figure 1 and Figure 2). The area under the curve (AUC) was 0.706 (CI: 0.581–0.830) for ICAM-1 and 0.731 (CI: 0.608–0.854) for IDREI. Figure 3 displays a box plot illustrating the distribution of IDREI values across the different study groups.

The optimal cut-off point for the IDREI variable, specifically for the DRE category, was determined using the Youden index. This calculation yielded a value of 34.53, which corresponded to a sensitivity of 63.2% and a specificity of 77.8%.

### 3.5. Multivariate Analysis

A multivariate logistic regression model was constructed to assess the independent contributions of selected variables. This model incorporated all statistically significant variables identified in preliminary analyses alongside variables deemed clinically relevant, with particular emphasis on those components of the IDREI. The outcomes of this multivariate analysis are presented in Table 5. The model demonstrated a robust fit, evidenced by a Nagelkerke’s Pseudo R-squared value of 0.543, indicating a substantial proportion of explained variance in the dependent variable.

**Figure 4:**
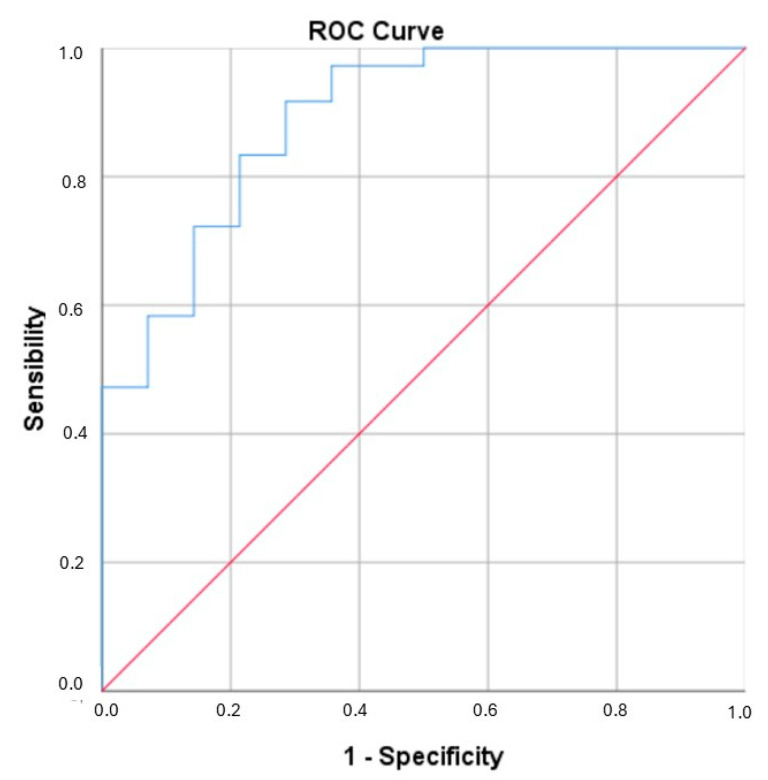
ROC for multivariate model. AUC of 0.891 (95% CI: 0.791–0.991).

Once the model was built, a ROC Curve was constructed for this model, which obtained an AUC of 0.891 (CI: 0.791–0.991) (Figure 4).

## 4. Discussion

Our investigation reveals that patients diagnosed with epilepsy, and more specifically those with DRE, exhibit a notable imbalance in circulating pro-inflammatory and anti-inflammatory factors. Among the panel of molecules analyzed in plasma, ICAM-1 emerged as the sole biomarker demonstrating statistical significance in both univariate and multivariate analyses. This finding underscores the potential centrality of ICAM-1 in the inflammatory dysregulation observed in these patient populations.

ICAM-1, a transmembrane glycoprotein, plays a crucial role in the immune system, particularly in mediating leukocyte recruitment and extravasation to sites of inflammation [20]. Its expression, typically low under basal conditions, is rapidly upregulated on endothelial cells in response to various inflammatory mediators, including cytokines like TNF-α and IL-1β [21]. This increased expression facilitates the adhesion and transmigration of leukocytes across the blood/brain barrier (BBB), a critical step in neuroinflammatory processes [22].

In the context of epilepsy, neuroinflammation and BBB dysfunction are increasingly recognized as key mechanisms contributing to epileptogenesis [23,24]. While some studies have not found significant differences in serum ICAM-1 levels in interictal epilepsy patients compared to controls [25], others suggest an upregulation of ICAM-1 and other adhesion molecules (e.g., VCAM) in epileptic patients, indicating increased BBB permeability [26]. Animal models of epilepsy have further demonstrated that seizures induce elevated expression of vascular cell adhesion molecules and enhance leukocyte rolling and arrest in brain vessels. Importantly, inhibition of these leukocyte/vascular interactions has been shown to reduce seizures and prevent BBB damage, highlighting a pathogenetic link between leukocyte/endothelial adhesion and seizure generation [27]. Thus, ICAM-1’s involvement in regulating leukocyte trafficking and potentially influencing BBB integrity positions it as a significant contributor to the complex neuroinflammatory cascade observed in patients with epilepsy, potentially impacting disease progression and severity.

For enhanced diagnostic precision in epilepsy, particularly in DRE, the development of an index reflecting the nuanced inflammatory balance is crucial. Such an index should integrate both pro-inflammatory and anti-inflammatory molecular markers. Based on our findings, we propose an index incorporating molecules with demonstrated pro-inflammatory potential alongside those exhibiting an anti-inflammatory profile that achieved statistical significance or approached it. Specifically, this latter group includes ICAM-1, NfL, IL-4, and IL-10.

NfL is a crucial biomarker of neuronal damage, reflecting axonal injury when released into the CSF and blood [28]. NfL itself, in addition, might contribute to neuroinflammation by activating immune cells like microglia, creating a self-perpetuating cycle of damage and inflammation [29]. Furthermore, NfL might help identify underlying mechanisms in refractory epilepsy, with some studies suggesting a correlation between NfL levels and cognitive impairment in epileptic patients [30].

From a neurobiological perspective, including a CSF biomarker like NfL adds robustness to the index. CSF biomarkers reflect CNS activity, such as neurodegeneration and neuroinflammation, providing greater specificity and correlation with brain pathology, especially in neuroinflammatory and infectious diseases [18,31].

Various previously validated diagnostic indices incorporate biomarkers in both plasma and CSF, particularly in the study of neurodegenerative diseases such as Alzheimer’s. For instance, the CSF/plasma ratio of Aβ42 and Aβ40 is typically around 25:1 in healthy individuals, which helps identify changes in amyloid transport or metabolism between the central nervous system and the periphery [32]. Additionally, internal ratios like Aβ42/Aβ40 are utilized in both plasma and CSF; an increase in the ratio in plasma and a decrease in CSF can indicate amyloid pathology. The correlation between these ratios in both compartments may offer further diagnostic information [33]. These combined indexes provide a more thorough evaluation of neurodegenerative and neuroinflammatory conditions, addressing the limitations of individual biomarkers within a single compartment. Therefore, combining CSF NfL with specific plasma neuroinflammatory biomarkers could offer a more comprehensive understanding of the disease [34].

Anti-inflammatory cytokines such as IL-10 and IL-4 are key players in mitigating the detrimental effects of neuroinflammation and may offer therapeutic avenues for preventing or ameliorating epilepsy. IL-10, a potent immunoregulator, primarily inhibits the synthesis of pro-inflammatory cytokines, suppresses T cell proliferation, and modulates microglial activation, thereby limiting neuronal damage and excitotoxicity often associated with seizures [35]. Similarly, IL-4, often associated with a T-helper 2 (Th2) immune response, can shift microglial phenotypes toward an anti-inflammatory, neuroprotective state, promoting tissue repair and reducing oxidative stress, both critical in preventing aberrant neuronal hyperexcitability [36].

The interrelationship between the levels of these 4 biomarkers can offer us an idea of the stage of neuroinflammatory balance higher than that of each molecule separately, thus improving our diagnostic capacity for DRE.

We propose the Inflammatory Drug-Resistant Epilepsy Index (IDREI), a novel biomarker derived from plasma concentrations of ICAM-1, IL-4, and IL-10 and CSF levels of NfL, for the prediction of DRE. Our study findings indicate that the IDREI demonstrates a 73.1% (95% CI: 70.8–85.4%) probability of accurately classifying patients with DRE. An optimal cut-off point of 34.53 for the IDREI yielded a sensitivity of 63.2% and a specificity of 77.8%, suggesting a moderate-to-good discriminatory capacity.

Furthermore, the predictive power of the model was significantly enhanced through multivariate analysis, incorporating both clinical and demographic data alongside the biological parameters. This comprehensive approach improved the predictive capacity to 0.891 (95% CI: 0.791–0.991), indicating a very good performance of the refined predictive model.

Our findings may have therapeutic applications in addition to their diagnostic value. While our study does not provide definitive conclusions regarding the pathogenic roles of biomarkers, further investigation into potential therapeutic interventions at this level is warranted. There are precedents where specific biomarkers have evolved into therapeutic targets, such as the use of anti-IL1 drugs like Anakinra in FIRES syndrome [37] or anti-TNFα in Rasmussen’s encephalitis [38].

Our study acknowledges several limitations, primarily concerning the small sample size, which may compromise statistical power, and its retrospective design. Furthermore, the integration of both plasma and CSF biomarkers presents a methodological challenge in clinical application. While recent advancements enable reliable plasma NfL determinations, the sustained validity of the index when utilizing plasma NfL requires specific validation.

## 5. Conclusions

Timely and precise identification of drug resistance is crucial for optimizing therapeutic strategies in the management of epilepsy. Numerous neuroinflammatory molecular biomarkers are intricately involved in the fundamental processes of epileptogenesis, and they hold significant promise as prognostic indicators. These biomarkers may enable the classification of patients based on their anticipated response to ASMs.

Often, the integration of multiple biomarkers can yield superior discriminative capacity compared to individual markers. This enhanced utility stems from their ability to collectively reflect the nuanced balance of neuroinflammation within the patient. The IDREI serves as a compelling example of such types of composite indices. Ultimately, the prospective clinical utility of the IDREI necessitates rigorous corroboration through well-designed prospective investigations.

## Figures and Tables

**Table 1:** Clinical and demographic variables. MRI: magnetic resonance imaging. EEG: electroencephalografy. IED: interictal epileptiform discharges. SV2A: synaptic vesicle glycoprotein 2A modulators.

		HC (*n* = 16)	SFE (*n* = 14)	DRE (*n* = 38)	*p*
Sex (%)	Male	31.25 (5/16)	42.85 (6/14)	39.47 (15/38)	0.78
	Female	68.75 (11/16)	57.15 (8/14)	60.52 (23/38)	
Age (±SD)		47.44 (19/94)	54.40 (15/70)	52.08 (14/22)	0.46
Clinical onset (±SD)		N/A	37.21 (21/18)	27.89 (15/35)	0.09
Duration of epilepsy (±SD)		N/A	16.29 (15/71)	23.39 (13/44)	0.11
Type of seizures (%)	Focal Seizures	N/A	78.57 (11/14)	42.10 (16/38)	0.147
	Focal and Bilteral tonico-clonic seizures	N/A	21.42 (3/14)	57.89 (22/38)	
MRI findings (%)	Normal	N/A	78.57 (11/14)	52.63 (20/38)	0.083
	Abnormal	N/A	21.42 (3/14)	47.36 (18/38)	
EEG findings (%)	Focal IED	N/A	42.85 (6/14)	65.79 (25/38)	**0.007**
	Multifocal IED	N/A	7.14 (1/14)	23.68 (9/38)	
	No IED	N/A	50 (7/14)	10.52 (4/38)	
Etiology (%)	Hippocampal sclerosis	N/A	21.42 (3/14)	28.9 (11/38)	0.082
	Focal Cortical Dysplasia	N/A	0 (0/14)	10.52 (4/38)	
	Gliotic lesion	N/A	0 (0/14)	5.26 (2/38)	
	Encephalocele	N/A	0 (0/14)	2.63 (1/38)	
	Non lessional	N/A	78.57 (11/14)	52.63 (20/38)	
Seizure frequency	Daily	N/A	0 (0/14)	26.31 (10/38)	**<0.001**
	Weekly	N/A	0 (0/14)	36.84 (14/38)	
	Monthly	N/A	0 (0/14)	21.05 (8/38)	
	Annual	N/A	0 (0/14)	15.78 (6/38)	
Concurrent ASMs (±SD)		N/A	1.57 (0/94)	2.55 (0/86)	**0.002**
Mechanism of action of ASM (%)	Sodium channel blockers	N/A	71.42 (10/14)	89.47 (34/38)	0.081
	Gabaergic	N/A	28.57 (4/14)	36.84 (14/38)	
	SV2A	N/A	28.57 (4/14)	55.26 (21/38)	

**Table 2:** Comparison of Levels of Biomarkers by Group. SD: Standard Deviation. IQR: Interquartile range. All determinations are expressed in pg/mL.

	Mean (SD)			Median (IQR)			*p*
	HC (*n* = 16)	SFE (*n* = 14)	DRE (*n* = 38)	HC (*n* = 16)	SFE (*n* = 14)	DRE (*n* = 38)	
**Plasmatic biomarkers**							
MIP-1a (CCL3)	2.37 (3.16)	6.20 (12.92)	3.83 (5.87)	1.12 (2.00)	2.62 (4.27)	1.73 (2.39)	0.318
IL-1b	9.82 (8.51)	5.27 (2.78)	6.98 (4.50)	6.42 (8.53)	4.02 (4.22)	5.85 (6.78)	0.220
IL4	12.37 (9.42)	7.64 (2.76)	8.50 (4.15)	9.11 (7.90)	7.71 (4.15)	8.61 (5.48)	0.169
IP-10 (CXCL10)	2.28 (1.57)	3.62 (2.33)	3.10 (1.99)	1.89 (2.11)	3.08 (4.48)	2.39 (3.50)	0.161
IL8 (CXCL8)	1.40 (0.24)	1.10 (0.53)	1.24 (0.54)	1.40 (0.41)	1.21 (1.18)	1.23 (0.54)	0.294
IL10	1.97 (1.42)	1.12 (0.63)	1.43 (0.90)	1.55 (1.65)	0.97 (0.97)	1.31 (1.34)	0.116
IL12	40.60 (29.96)	20.72 (14.24)	28.59 (21.69)	39.53 (37.46)	13.43 (27.46)	27.38 (33.89)	0.095
IL13	9.42 (5.65)	9.55 (4.79)	8.73 (4.99)	6.08 (7.15)	11.29 (8.46)	7.24 (6.60)	0.636
IL17A	6.94 (3.34)	7.06 (3.15)	6.98 (2.75)	6.40 (4.44)	6.52 (5.19)	6.19 (2.15)	0.971
IL33	4.73 (2.85)	3.53 (1.15)	4.42 (2.87)	3.90 (4.06)	3.50 (1.89)	3.49 (3.35)	0.663
IFN-g	2.87 (3.91)	6.64 (5.51)	5.41 (5.79)	1.05 (1.59)	8.02 (10.73)	1.50 (33.56)	0.181
GM-CSF	54.20 (31.67)	34.50 (24.57)	42.47 (31.49)	48.88 (38.58)	23.16 (48.54)	36.50 (43.18)	0.160
TNF-a	13.81 (6.24)	14.80 (6.84)	15.65 (6.84)	12.76 (9.02)	13.32 (9.00)	14.99 (8.80)	0.504
MIP1-b	11.96 (4.60)	12.04 (4.63)	14.45 (11.56)	10.50 (8.63)	10.02 (7.02)	11.00 (5.90)	0.375
IFN-a	1.03 (0.84)	1.42 (0.80)	1.20 (0.73)	0.72 (1.25)	1.32 (1.28)	0.95 (1.06)	0.422
MCP-1	10.34 (6.08)	17.95 (11.08)	13.96 (8.26)	9.64 (8.14)	18.34 (17.40)	11.42 (11.49)	0.145
P-SELECTINE	16,005.10 (39,258.50)	17,186.03 (9084.97)	23,688.20 (46,499.29)	10,260.24 (12,498.80)	20,237.49 (18,099.64)	13,435.29 (14,714.05)	0.538
IL1-a	0.57 (0.37)	0.41 (0.24)	0.68 (1.14)	0.48 (0.54)	0.32 (0.34)	0.50 (0.31)	0.285
**ICAM-1**	**35,193.99 (118,445.28)**	**50,128.29 (34,555.28)**	**351,519.10 (983,829.16)**	**33,781.59 (92,659.30)**	**43,290.99 (40,665.20)**	**67,975.41 (170,840.08)**	**0.035**
E-SELECTINE	5442.02 (3718.05)	6659.13 (4264.43)	6268.81 (4044.53)	5913.69 (5589.29)	6946.73 (7445.26)	5560.47 (7085.13)	0.809
rsTNF-RII	2083.04 (789.64)	2370.70 (476.90)	2368.34 (767.92)	1719.94 (845.10)	2424.01 (759.34)	2420.43 (1089.06)	0.271
TRL4	2323.72 (1404.43)	2777.24 (3053.35)	2421.90 (2070.78)	2043.52 (1963.93)	1705.35 (1236.20)	1883.44 (1384.76)	0.804
HMGB	9919.10 (11,223.93)	9385.59 (7822.42)	6870.22 (3727)	6586.39 (7592.98)	6635.64 (4578.25)	6121.22 (3891.71)	0.442
**CSF biomarker**							
NfL	391.61 (317.45)	683.09 (693.19)	596.82 (617.01)	236.10 (343.15)	398.99 (501.51)	462.77 (415.50)	0.145

**Table 3:** Comparison of IDREI values by group.

Index	Median (IQR)			*p*
	HC (*n* = 16)	SFE (*n* = 14)	DRE (*n* = 38)	
**IDREI**	4.46 (31.80)	25.53 (70.62)	64.10 (87.58)	**0.002**

**Table 4:** Collinearity analysis for variables included in IDREI.

Variable	Tolerance	VIF
**IL4**	0.611	1.636
**IL10**	0.603	1.657
**ICAM-1**	0.981	1.020
**NfL**	0.997	1.003

**Table 5:** Multivariate model.

	Chi-Square	*p*
Concomitant ASM	3.357	0.067
Clinical onset	0.756	0.385
IL10	1.304	0.254
**ICAM-1**	**5.047**	**0.025**
NfL	0.530	0.467
IL4	1.026	0.311
EEG findings	1.692	0.429
Type of seizures	0.014	0.906

## Data Availability

The data presented in this study are available on request from thecorresponding author due to ongoing research studies that use the same database.

## Supplementary Materials

The following are available online at https://www.mdpi.com/article/10.3390/biom15070914/s1.

## Abbreviations

The following abbreviations are used in this manuscript:

DRE	Drug-resistant epilepsy
SFE	Seizure-free epilepsy
HC	Healthy control
IDREI	Inflammatory Drug-Resistant Epilepsy Index
AUC	Area under the curve
ROC	Receiver operating characteristic
CI	Confidence interval
IQR	Interquartile range
WHO	World Health Organization
ASM	Anti-seizure medications
ILAE	International League Against Epilepsy
MIP-1α	Macrophage inflammatory protein 1-alpha
IL-1β	Interleukin-1 beta
IP-10	Interferon gamma-induced protein 10 or CXCL10
IL-8	Interleukin-8
IL-12	Interleukin-12
IL-17A	Interleukin-17A
IL-33	Interleukin-33
IFN-γ	Interferon gamma
GM-CSF	Granulocyte-macrophage colony-stimulating factor
TNF-α	Tumor necrosis factor-alpha
MIP-1β	Macrophage inflammatory protein 1-beta
IFN-α	Interferon-alfa
MCP-1	Monocyte chemoattractant protein-1
IL-1α	Interleukin-1 alfa
ICAM-1	Intercellular adhesion molecule-1
sTNF-RII	Soluble tumor necrosis factor receptor TNF-RII
TLR4	Toll-like receptor 4
HMGB1	High Mobility Group Box 1
IL-4	Interleukin-4
IL-10	Interleukin-10
IL-13	Interleukin-13
NfL	Neurofilament Light Chain
ELISA	Enzyme-linked immunosorbent assay
MRI	Magnetic resonance imaging
EEG	Electroencephalography
ANOVA	Analysis of variance
SV2A	Synaptic vesicle glycoprotein 2A modulators
SD	Standard deviation
IED	Interictal epileptiform discharges.
VIF	Variance Inflation Factor
BBB	Blood/brain barrier
VCAM	Vascular cell adhesion molecule
Aβ42	42-amino acid peptide fragment of the amyloid precursor protein
Aβ40	40-amino acid peptide fragment of the amyloid precursor protein
