# Supplementary material for: Inflammatory Drug-Resistant Epilepsy Index (IDREI) as a Molecular Compound Biomarker in Focal Epilepsies
Source: Biomolecules. 2025 Jun 22;15(7):914. doi: 10.3390/biom15070914 (PMC12292844; doi:10.3390/biom15070914)
Supplement: Supplementary file 1 [file biomolecules-15-00914-s001.zip › biomolecules-3694432-supplementary.pdf]

Detailed biomarker detection procedure:

2.4.2. Multiplex Assay

Inflammatory and Anti-inflammatory factors were evaluated in plasma samples using a ProcartaPlex™ Human Inflammation Panel, 20plex kit (catalogue number: EPX200-12185-901; Thermo Fisher Scientific Inc., Waltham, MA, USA). Wash Buffer Concentrate (10x) required vortex and dilution in double-distilled water for 1x solution. Lyophilized multistandards containing the mix of standard proteins were reconstituted to 250 μL with provided Universal Assay Buffer (1x) and used for a 1:4 serial dilution for a standard 7-point curve. The same buffer was used for blank and plasma samples (1:2). As the first step of the assay the Magnetic Bead solution was vortexed for 30 s and 50 μL were dispensed into each assay well for standards and samples of a 96-well Flat Bottom Plate inserted into a Hand Held Magnetic Plate Washer. Beads settled for 2 min before the liquid was decanted. For washing, 150 μL of wash buffer was added to each well followed by a 30-minute incubation. After decanting, the plate was gently pressed against absorbent paper to remove residual liquid, and the plate was detached from the magnetic plate washer. After that, 50 μL of appropriately diluted standards and experimental samples were added to the designated wells and run in duplicate. The plate was sealed, covered with a black microplate lid to protect from light, and shaken at 500 rpm for 120 minutes at room temperature. Wash steps were performed as described above. After washing (2x), 25 μL of the detection antibody mix was added to each well. The plate was sealed, covered and shaken, as described, for 30 min at room temperature. Following incubation, the plate was washed (2x). Afterward, 50 μL of streptavidin-phycoerythrin (SAPE) solution was added to enable fluorescent labeling of the analyte-bound complexes. The plate was sealed, covered and shaken, as previously, at 500 rpm for 30 min at room temperature. After washing (2x) 120 μL of Reading Buffer was added to each well. The plate was sealed, covered and shaken at 500 rpm for 5 min at room temperature. Then, the cover and seal were removed, and the plate was run for detection using xMAP® technology on the Luminex MAGPIX® instrument (Diasorin S.p.A., Saluggia VC, Italy). Acquisition and analyses were performed using xPONENT® 4.3 software (Diasorin S.p.A., Saluggia VC, Italy).

2.4.3. ELISA Assay

Samples were diluted according to the requirements of the corresponding kits. Duplications were made for each sample. Analyses were performed following the manufacturers' standard protocols, with appropriate quality controls to ensure the accuracy and reliability of the results. Quantification of HMGB1 and TLR4 was performed using an ELISA kit from ElabScience® (Houston, TX, USA), with catalogue numbers E-EL-H1554 and E-EL-H1539, respectively. Similarly, samples were tested for sTNFR II using a specific ELISA kit (Human sTNF RII/TNFRSF1B Quantikine® ELISA Kit DRT200; R&D Systems, Inc., Bio-Techne Ltd., Minneapolis, MN, USA).

For HMGB1 and TLR4 ELISA kits, 100 μL of the appropriately diluted standards and samples were added to the microplate wells. The plate was then sealed and incubated for 90 minutes at 37°C. The calibration curve used serial 1:2 dilutions. The dilution of the standards and plasma samples (1:4) were made using the supplied sample dilution buffer. After discarding the liquid without washing, 100 μL of the biotinylated detection antibody working solution was added to each well and incubated for 60 min at 37 °C. After incubation, the liquid was aspirated, and the plate was washed three times by adding 350 μL of wash buffer to each well. After decanting, the plate was gently pressed against absorbent paper to remove residual liquid. Next, 100 μL of the working avidin-HRP conjugate solution was added to each well of the plate, which was then incubated for 30 minutes at 37°C after sealing. Then, the plate was washed five times as previously described and 90 μL of substrate reagent was added to each well. The plate was sealed and covered from light, then incubated for 15 min at 37°C. Finally, 50 μL of stop solution was added to each well, and the optical density (OD) was measured immediately at 450 nm using an ELISA reader (Lan Technics Model R-100, Hangzhou Allsheng Instruments Co., Ltd., Hangzhou, China).

Regarding the sTNF RII/TNFRSF1B assay, samples required a 10-fold dilution in provided Calibrator Diluent RD6O, which was also used for the standard curve. 50 µL of Assay Diluent RD1-6 was added to each well. After mixing thoroughly, 200 µL of standard or sample were dispensed per well. The plate was then sealed and incubated at room temperature for 2 hours. Three washing steps were performed as previously described. Next, 200 µL of Human TNF RII Conjugate was added to each well and the plate was sealed and incubated at room temperature for another 2 hours. Following incubation, the plate was washed (3x) and 200 µL of Substrate Solution was added to each well. The plate was then sealed, protected from the light, and incubated for 2 hours at room temperature. Finally, 50 µL of stop solution was added to each well, and the OD was measured as previously described.

The UmanDiagnostics NF-light™ ELISA RUO kit (10-7002, Quanterix Corp., Billerica, MA, USA) was used for NfL measurement in CSF. CSF samples were diluted with equal volume of supplied sample diluent, ensuring a minimum total volume of 210 µL. The standards (8 points, including blank) were reconstituted and diluted following the manufacturer’s instructions. Before sample addition, the wells were washed three times by pipetting 300 µL of wash buffer. Then, 100 µL of each standard or sample were added in duplicate. The plate was incubated at room temperature for 1 hour with agitation (800 rpm). After washing, 100 µL of freshly diluted Tracer biotin-labeled antibody (1:50 in sample diluent) was added to each well and incubated at room temperature for 45 min with agitation (800 rpm). The plate was washed again, and 100 µL of freshly diluted Streptavidine-HRP Conjugate (in supplied diluent) was added to each well, followed by 30 min of incubation at room temperature with agitation (800 rpm).

After incubation, the plate was washed, and 100 µL of TMB substrate solution was added to each well. The plate was then protected from the light and incubated for 15 min at room temperature. Finally, 50 µL of stop reagent solution was added to each well, and the OD was measured as previously described.

Samples were diluted according to the requirements of the corresponding kits. Duplications were made for each sample.

Analyses were performed following the manufacturers' standard protocols, with appropriate quality controls to ensure the accuracy and reliability of the results.
